# Myocardial fibrosis and inflammation after stereotactic ablative body radiotherapy for ventricular tachycardia associated with apical left ventricular aneurysm in hypertrophic cardiomyopathy

**DOI:** 10.1016/j.hrcr.2025.07.026

**Published:** 2025-08-05

**Authors:** Johanna B. Tonko, Christopher Dean, Edd MacLean, Nick Plowman, Pier D. Lambiase, Mehul Dhinoja, Anish Naresh Bhuva

**Affiliations:** 1 Institute for Cardiovascular Science, University College London, London, United Kingdom; 2 Department for Cardiology, St Bartholomew’s Hospital, London, United Kingdom; 3 Radiotherapy Physics, St Bartholomew’s Hospital, London, United Kingdom

**Keywords:** Ventricular tachycardia, Radiation therapy, Ablation, Hypertrophic cardiomyopathy, Apical aneurysm, Myocardial remodeling, SABR

**Key Teaching Point**

• Stereotactic ablative body radiotherapy (SABR, 25 Gy) appears to be safe and effective in suppressing monomorphic ventricular tachycardia (VT) arising from an apical aneurysm in a patient with hypertrophic cardiomyopathy.
• The therapeutic mechanism of VT suppression after SABR likely involves progression and homogenization of fibrosis and myocardial inflammation.
• The case report supports the potential role of SABR as a non-invasive therapeutic option for recurrent VT in patients with aneurysmatic scar and high procedural risk for conventional catheter-based or surgical ablation.

## Introduction

Cardiac stereotactic ablative body radiotherapy (SABR) (also known as “stereotactic arrhythmia radioablation [STAR]”) is an emerging non-invasive ablation approach for scar-related ventricular tachycardia (VT), which delivers an obliterative dose of targeted radiation to the functionally critical areas of the VT circuit. This makes it potentially attractive in patients with high procedural risk for conventional invasive VT ablation, and can overcome anatomical constraints to reach otherwise inaccessible target zones (ie, deep intramural substrate or subepicardial substrate in conditions precluding epicardial access). Its success in the treatment of refractory VT has been moderated by reports of recurrence after 6 months.^1^ In addition, most outcome data are limited to patients with ischemic or dilated cardiomyopathy.^2^^,^^3^ SABR use for hypertrophic cardiomyopathy (HCM)-related VT is sparse, and safety of the radiation of thinned-out aneurysmal areas is not well-defined. In addition, the time course and mechanisms of the clinical and myocardial response to cardiac radio-ablation are still not identified, but may be due to initial changes in sodium channel activity^4^ and then the development of dense myocardial fibrosis.^5^ Cardiovascular magnetic resonance (CMR) imaging can provide a non-invasive substrate assessment and evaluate architectural tissue changes after treatment.

We present a case of an 86-year-old patient with HCM with recurrent hospitalization for drug-refractory VT associated with a large apical aneurysm undergoing SABR using the Accuray CyberKnife platform due to prohibitive risk for conventional catheter ablation. We report detailed scar characterization from both before and after SABR-ablation using CMR.

## Case report

An 86-year-old patient with known HCM and apical aneurysm was referred for evaluation of urgent catheter ablation following recurrent drug-refractory monomorphic VT and background of amiodarone-induced pulmonary fibrosis.

### Background

The patient had a longstanding diagnosis of HCM with severe left ventricular (LV) impairment. A cardiac resynchronization therapy pacemaker (CRT-P) had been previously inserted in the context of a pace-and-ablate strategy for permanent atrial fibrillation. Two years after CRT-P insertion, the patient presented with an electrical storm with a total of 14 syncopal VT episodes requiring external cardioversion. Her CRT-P was upgraded to a CRT defibrillator (CRT-D). Medical therapy with sotalol, and then in combination with mexiletine, failed to suppress recurrent VT with implantable cardioverter defibrillator (ICD) therapies, resulting in repeated hospitalizations and debilitating drug-related side effects.

**Figure 1:**
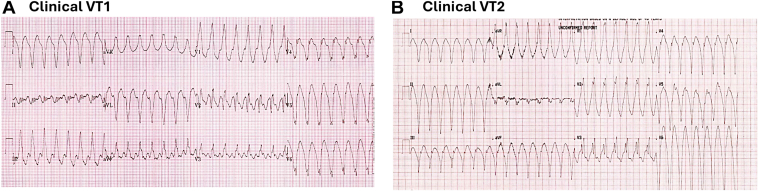
12-lead ECGs of clinical VTs. ECG = electrocardiogram; VT = ventricular tachycardia.

A 12-lead electrocardiogram (ECG) of the spontaneous clinical VT documented 2 different morphologies, with right bundle morphology and precordial transition in V4, one with superior axis and one with inferior-rightward axis (see Figure 1).

The patient had a small body size (height 145 cm, weight 45 kg), was frail (Rockwood frailty scale 6, World Health Organization performance status 3), and had numerous comorbidities (see Table 1). The periprocedural risk of a conventional invasive VT ablation was deemed prohibitive. Given the high burden of VT with recurrent ICD therapies and hospitalization, the patient was recruited into a prospective feasibility trial (SABRE-VT Trial, ClinicalTrials.gov ID NCT05696522) to improve quality of life. Although no formal exemption for publication was sought, the investigator team felt that the unique clinical features of this case warranted a separate report to allow a detailed description of the treatment planning, delivery, and outcome.

**Table 1:** Comorbidities and medication (pre-SABR)

A. Comorbidities
Frailty (Rockwood 6)
Permanent AT/AF
Moderate AR & moderate-severe MR
Pulmonary fibrosis (amiodarone)
Previous left-parietal CVA
Hypothyroidism
Obstructive bile duct stones
Left subclavian occlusion
B. Medication
Apixaban 2.5 mg BD
Sotalol 120 mg TDS
Mexiletine 100 mg TDS
Candesartan 4 mg OD
Thyroxine 25 μg OD
Atorvastatin 40 mg OD
Pregabalin 50 mg OD
Amitriptyline 10 mg OD

AR = aortic regurgitation; AT/AF = atrial tachycardia/atrial fibrillation; BD = twice a day; CVA = cerebrovascular accident; MR = mitral regurgitation; OD = once a day; SABR = stereotactic ablative body radiotherapy; TDS = three times a day.

### SABR-planning

As per the institutional protocol, the patient underwent a CMR, contrast-enhanced cardiac and radiotherapy planning computed tomography (CT) scans, and non-invasive VT induction via her ICD with ECG imaging (ECGI) performed for electrophysiological target definition.

**Figure 2:**
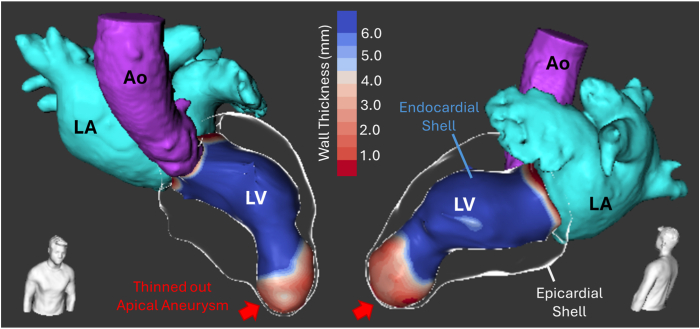
Pre-procedural wall thickness CT model (visualized with Adas3D, Galgo Medical). Ao = aorta; CT = computed tomography; LA = left atrial; LV = left ventricular.

**Figure 3:**
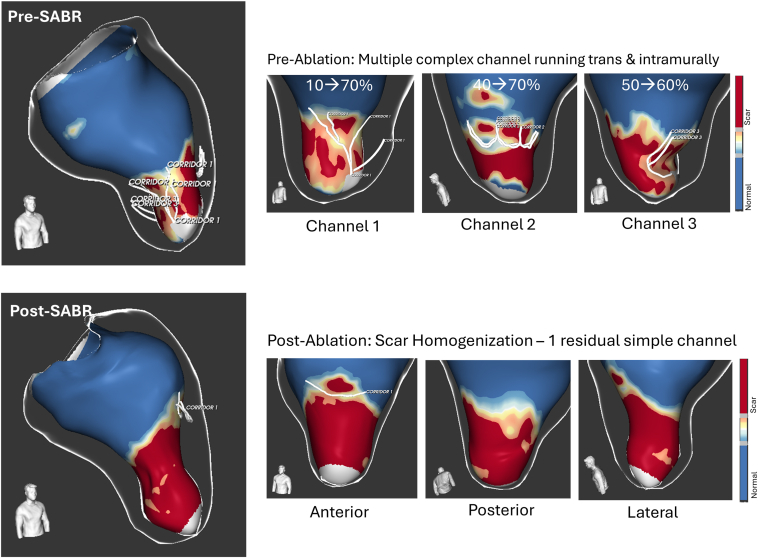
3D DE-LV model pre- and post-SABR. *Top*: The pre-procedure CMR model revealed heterogenous scar with numerous complex channels with various side branches and transecting multiple myocardial layers (% indicate the transmural depth at which each channel was identified, 10% [subendocardial] to 90% [subepicardial]). *Bottom*: The post-procedure CMR models demonstrate an overall more extensive, but more homogenized scar area with only one residual simple channel at the anterior edge of the scar can be identified. 3D = 3 dimensional; DE-LV model = delayed enhancement left ventricular model; SABR = stereotactic ablative body radiotherapy.

A reconstructed 3-dimensional (3D) CT wall thickness model (Adas 3D, Galgo Medical, Barcelona, Spain) showed a large thinned-out apical aneurysm and compressed midventricular segments (Figure 2). The CMR demonstrated asymmetric mid-cavity hypertrophy (maximal wall thickness 20 mm in the mid-anteroseptal segment), with a large apical aneurysm with circumferential partially transmural scar involving the apical cap and extending inferiorly in the midventricular segment. LV ejection fraction (LVEF) was 34%. 3D models of the late gadolinium enhancement (LGE) sequence revealed a heterogeneous scar architecture, with multiple conducting channels around the aneurysm neck (Figure 3, Top Panel).

**Figure 4:**
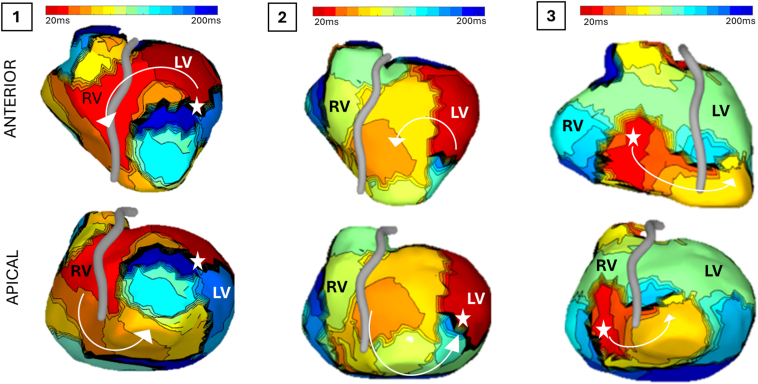
Isochronal ECGI activation maps of NIPS-induced VTs. VT activation maps following VT induction with non-invasive VT stimulation (Panel 1-3): *Red* indicates the earliest activation on reconstructed unipolar EGMs, representing the presumed VT exit and locating to variable sites around the aneurysmal neck, including sites consistent with the spontaneous VT morphologies captured on 12-lead ECG. ECGI acquired with CardioInsight^TM^ (Medtronic). ECG = electrocardiogram; ECGI = electrocardiogram imaging; EGM = electrogram; LV = left ventricular; NIPS = non-invasive programmed stimulation; RV = right ventricular; VT = ventricular tachycardia.

Programmed ventricular stimulation was delivered from the right ventricular lead using a standardized protocol, with an 8-beat drive train with constant S1-interval (600 ms), followed by up to 3 progressively decrementing sensed extra beats. The protocol was then repeated with a 400 ms drive, which successfully induced monomorphic VT, allowing for detailed activation mapping with ECGI and subsequent pace-termination. Non-invasive ECGI mapping suggested multiple exit sites around the anterior aneurysmal neck (VT activation maps illustrated in Figure 4). Activation maps 1 and 2 were in agreement with the exit site of the spontaneous VT1 as suggested by the 12-lead surface ECG morphology, but VT2 was not seen during ECGI mapping.

As per national consensus, the clinical history, investigations, and planning were discussed for eligibility, target zone identification, and treatment planning at regular national United Kingdom cardiac SABR meetings.

### SABR delivery

**Figure 5:**
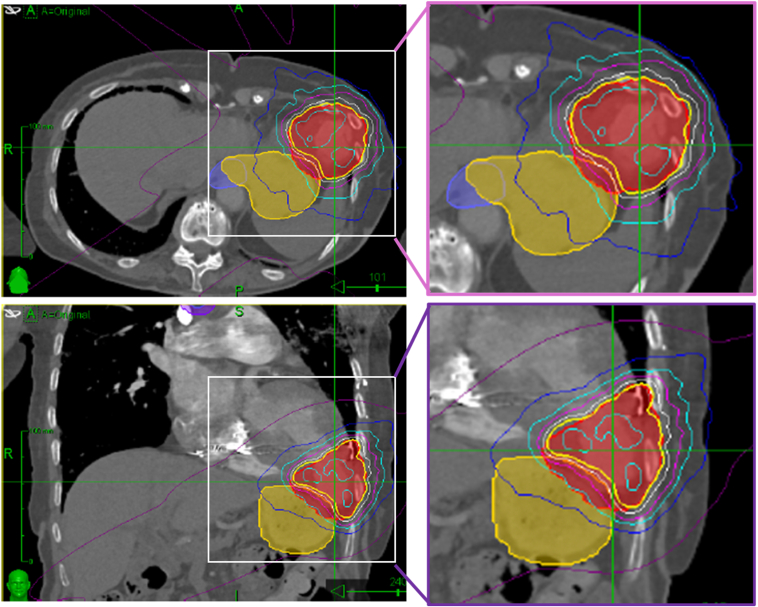
Planning CT with isodose areas. *Red volume* = PTV, *Yellow line* = 25 Gy isodose focused on cardiac target area; *Yellow area* = stomach. The target area was designed to encompass the aneurysm, its neck, and adjacent midventricular segments to include the entire scar border zone, which on the endocardium extended into the midventricular segments. These regions were targeted to homogenize the heterogeneous tissue architecture associated with the border zone, thereby also promoting scar progression toward the epicardial layer at these sites and contributing to an overall increase in the scar burden. CT = computed tomography; PTV = planning target volume.

Gross target volume (GTV) was delineated on the 3D radiotherapy planning CT scan (Varian Eclipse, v.15) after co-registering all additional imaging described above. The GTV incorporated the entire aneurysm and its neck, and was extended to include the entire border zone defined by LGE-magnetic resonance imaging (MRI) and gradual thinning of the myocardial wall on CT (GTV 44.9 cm^3^). Although the CyberKnife Synchrony system was used to track the intracardiac lead tip close to the target, as per usual protocol, the GTV was expanded to an internal target volume (ITV) to account for any additional cardiac and respiratory motion using the 4-dimensional (4D) respiratory binned planning CT data and MRI data. An ITV-planning target volume (PTV) margin was then added to account for uncertainty in on-treatment localization, image co-registration process, and the dose calculation, generating a final PTV of 100.7 cm^3^. Treatment planning was performed using inverse-planning with the Accuray VOLO optimizer (Accuray Precision®, V3.3.0.0) (Figure 5). Dose calculation was performed and dose constraints to off-target thoracic (including coronaries) and abdominal structures assessed. A dose of 25 Gy was prescribed to the surface of the PTV (73.2% effective prescription isodose, max dose 34.2 Gy). The plan respected all mandatory local organ at-risk dose tolerances, except the very distal coronary arteries (D0.01 cm^3^ = 24.8 Gy, tolerance dose 18.5 Gy). Yet, this deviation was intentional, as the consensus was in favor of prioritizing PTV coverage. The PTV coverage was only compromised in the apical region due to the proximity of the stomach and small bowel (a minimum of 25 Gy covering 82% of the PTV and 89.1% of the ITV). The prescribed dose was delivered as a single fraction without acute complications.

### Post-SABR follow-up and investigations

Five months after SABR delivery, the patient was weaned off Mexiletine. Sotalol was continued on a reduced dose of 120 mg twice daily. Over a total follow-up of 18 months, isolated non-sustained VTs, but no further sustained VT or ICD therapy, were documented.

A routine follow-up echocardiogram 4 months post-procedure reported a stable LVEF, no evidence of LV thrombus in the apical aneurysm, and no pericardial effusion. Pre-existing valve diseases were unchanged. A repeated echocardiogram 10 months post-procedure diagnosed a new circumferential pericardial effusion up to max. 17 mm on the anterior LV wall. In the absence of hemodynamic relevance or associated symptoms, this was managed conservatively.

**Figure 6:**
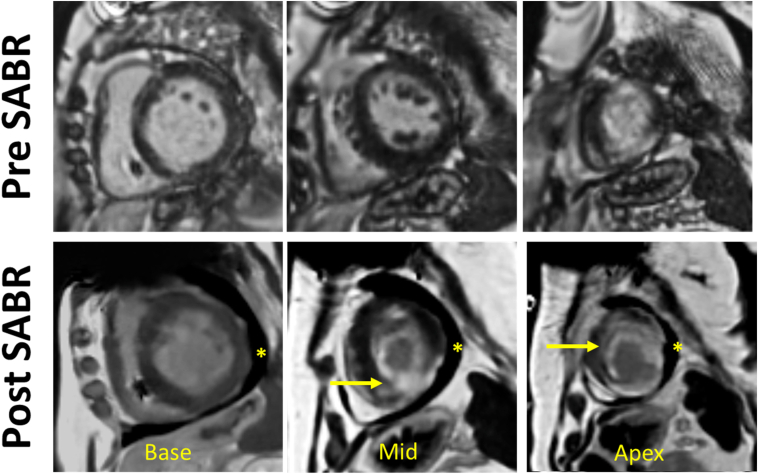
Baseline and follow-up cardiovascular magnetic resonance (CMR) at 12 months post-radiotherapy. Baseline CMR late gadolinium enhancement (LGE) short-axis imaging pre- (*top*) and 12 months post-radiotherapy (*bottom*). The pre-SABR baseline LGE revealed a patchy transmural apical scar progressing post-SABR to a more transmural and homogenous scar within the apical aneurysm and aneurysm neck (*arrows*). The post-SABR CMR also showed a new pericardial effusion (∗). SABR = stereotactic ablative body radiotherapy.

A repeated cardiac MRI scan 12 months after SABR showed stable LVEF. Pericardial effusion remained present (max. 13 mm adjacent to RA), and increased T1 and T2 relaxation suggested active myocardial inflammation. Visually, baseline patchy transmural apical LGE had progressed to a more transmural and homogenous pattern within the apical aneurysm and aneurysm neck (Figure 5, Figure 6 and Figure 5, Figure 6).

### Scar tissue characterization of pre- and post-MRI scans

Pre- and post-SABR delayed-enhancement MRI sequences were compared to quantify changes in scar burden, complexity, and presence of 3D channels (Adas3D^TM^ software, V2.14). Post-ablation, there was a significant increase in overall scar mass and area, with greater expansion on the epicardial surface compared to the endocardium. These changes likely reflect the underlying ventricular geometry and pathological substrate rather than preferential epicardial targeting by SABR. Specifically, the endocardial cavity was relatively small, whereas the corresponding epicardial surface was more expansive yet initially less scarred. The inclusion of the midventricular segments in the ablation target area encompassing the border zone of the endocardial scar, slightly beyond the aneurysm, contributed to the outward extension of scar formation and also the substantial increase in total scar mass. Sub-analysis of scar composition revealed a notable increase in dense scar (“core,” threshold: >60%) with only minimal change in border zone (thresholds: 40%–60%). The number and complexity of conductive channels (CCs) were reduced from 3 multilayer CCs to 1 simple CC. Tabulated results of tissue characterization are reported in Table 2. Side-by-side comparison of 3D models is shown in Figure 2, Figure 3, and Figure 2, Figure 3D slices are illustrated in Figure 6, demonstrating a more extensive but homogenized scar area, including in the apical region where prescription dose coverage was compromised due to proximity to the stomach and large bowel.

**Table 2:** Pre- and post-procedure scar tissue characterization

	PRE	POST	DIFF
Scar area endo (total area)	32.3 cm^2^ (139.6 cm^2^)	40.2 cm^2^ (125.2 cm^2^)	+7.9 cm^2^ (24.5%)
Scar area epi (total area)	36.0 cm^2^ (206.5 cm^2^)	59.5 cm^2^ (200.6 cm^2^)	+23.5 cm^2^ (65.2%)
Total scar mass - Core (>60%) - BZ (40%–60%)	26.7 g 11.2 g (41.9%) 15.5 g (58.1%)	49.4 g 32.5 g (65.8%) 16.9 g (34.2%)	+22.7 g (85.0%) +21.3 g (22.0%) +1.4 g (9.0%)
3D channels number - Channel length - Channel width	3 C1 length 69.7 mm Width 6.9 ± 2.3 mm C2 length 31.4 mm Width 6.5 ± 1.4 mm C3 length 23.4 mm Width 5.0 ± 2.3 mm	1 C1 length 19.7 mm Width 7.8 ± 1.1 mm	-2
Area of wall thinning <3 mm	6.7 cm^2^	6.8 cm^2^	+0.1 cm^2^

BZ = border zone; DIFF = difference; POST = post-procedure; PRE = pre-procedure.

## Discussion

Here, we present a case of SABR to treat drug-refractory scar-related VT originating from an apical aneurysm in HCM, including extended follow-up and repeated tissue characterization with cardiac MRI. Treatment was performed on the CyberKnife platform, tracking target myocardium using the LV lead tip as a fiducial marker (surrogate), and successfully suppressing recurrent sustained VT and ICD therapies during long-term follow-up. At 10 months post-procedure, an asymptomatic pericardial effusion was diagnosed. Repeated CMRI suggested therapeutic mechanisms may include scar homogenization and myocardial inflammation. There were no acute or long-term clinically significant adverse events.

Since the landmark publication of the first case series involving 5 patients with dramatic reduction in VT burden,^6^ further single- and multi-center case series^7^^,^^8^ and prospective studies^3^^,^^9^ have followed, and several SABR programs and trials are ongoing.^10^ Thus far, most experience relates to patients with ischemic and non-ischemic dilated cardiomyopathies, and there are only isolated case reports for arrhythmogenic cardiomyopathy and HCM.^11^ To our knowledge, there is only a single case describing SABR treatment for an apical aneurysm in burned-out HCM using a conventional linear accelerator,^12^ and thus there is an important need to provide further evidence within that patient cohort and application of CyberKnife as a treatment platform.

### Considerations for SABR therapy for VT in HCM with aneurysm

Apical aneurysm in HCM have been associated with an increase in morbidity and mortality, with a 3-fold higher event rate compared with patients with HCM without aneurysm, increased susceptibility to monomorphic VT, and a sudden death event rate of 4.7%/year.^13^ The overall prevalence of aneurysm in the HCM population is around 4%–5%; however, in patients with HCM with refractory VT, systematic reviews have described it as high as 32%.^14^ The pathophysiology of apical LV aneurysm formation in these patients has been reviewed,^15^ and a significant association between midventricular gradient and subsequent apical aneurysm formation has been described,^16^ as well as their association with significant LGE increase over time.^17^

Treatment of scar-related VT in association with ventricular aneurysm in HCM can be challenging, and frequently requires combined endo-epicardial procedures to achieve abolition of the arrhythmogenic substrate.^14^ Procedural success rates have been reported as high as 78% over an average of a 3-year follow-up in smaller case series employing this approach,^18^ at the cost of increased procedural complexity and complication rates. Lastly, anesthetic management in patients with HCM (obstructive) undergoing VT ablations, with the frequent need for pharmacological hemodynamic support, can be challenging.^19^

As such, these patients would represent the ideal population for non-invasive ablation by means of SABR, obviating the need for anesthetics and minimizing periprocedural risks while maintaining a high likelihood of achieving transmural lesion formation. In turn, there is justified concern that the thinned-out dyskinetic tissue of an aneurysm may present a higher risk for perforation, particularly if significant intracavitary pressure gradients remain and exert mechanical stress on the ablation area. In addition, evidence informing about optimal target dose and extent of target volume is very limited in these patients.

Our case report demonstrates that even in elderly, frail, and very small patients, the usual prescription dose of 25Gy can be delivered safely, with no serious clinically relevant adverse events and efficiently achieving a complete suppression of her sustained VTs over an 18-month follow-up period, compared to recurrent refractory VT with 3 hospitalizations in the 5 months preceding the treatment.

## Conclusion

SABR appears safe and effective in treating drug-refractory VT associated with apical aneurysm in HCM. This case suggests that therapeutic mechanisms may include myocardial inflammation and progression of fibrosis.
